# Seasonal Variation and Meteorological Correlates of Botulinum Toxin Injections for Axillary Hyperhidrosis in Japan: A Retrospective Analysis

**DOI:** 10.7759/cureus.83996

**Published:** 2025-05-12

**Authors:** Yoshiaki Kabata

**Affiliations:** 1 Ophthalmology, Jikei University School of Medicine, Daisan Hospital, Tokyo, JPN

**Keywords:** botulinum toxin, japan, ndb, primary axillary hyperhidrosis, season

## Abstract

Background: Primary axillary hyperhidrosis, characterized by excessive underarm sweating without an underlying medical condition, significantly impairs quality of life. While various treatments exist, botulinum toxin type A injections are widely used in Japan, especially for severe cases. Although increased sweating is commonly observed during warmer months, the relationship between treatment demand and meteorological factors remains unclear. This study aims to determine whether there are seasonal differences in the number of axillary hyperhidrosis injections in Japan and to investigate their relationship with meteorological conditions.

Methods: A retrospective, descriptive analysis was conducted using the National Database of Health Insurance Claims and Specific Health Checkups of Japan (NDB) from fiscal years 2019 to 2022. Monthly injection data were correlated with meteorological variables (temperature, precipitation, solar radiation, wind speed, relative humidity, atmospheric pressure) obtained from the Japan Meteorological Agency. Seasonal differences were assessed using the Steel-Dwass test, and partial correlation analysis examined associations between meteorological factors and injection numbers.

Results: The number of axillary hyperhidrosis injections peaked consistently in May and June each year, with significantly higher volumes in spring and summer compared to fall and winter (p<0.001). Partial correlation analysis revealed a strong positive correlation between all-day solar radiation and injection numbers (r=0.7193; p<0.0001), while temperature (r=-0.6052; p<0.0001) and wind speed (r=-0.441; p=0.0031) were negatively correlated. Relative humidity showed a moderate positive correlation (r=0.3626; p=0.0169). The seasonal peak preceded the hottest months, suggesting proactive treatment-seeking behavior.

Conclusions: Botulinum toxin injections for axillary hyperhidrosis in Japan display a reproducible seasonal pattern, with demand peaking in late spring and early summer. Solar radiation, rather than temperature alone, is most closely associated with treatment frequency, indicating that patients may seek care in anticipation of increased symptoms. These findings can inform healthcare resource planning and patient education to optimize the management of axillary hyperhidrosis.

## Introduction

Primary axillary hyperhidrosis is characterized by excessive sweating in the underarms that occurs without an identifiable underlying medical condition [1-4]. Studies conducted in Japan have estimated the prevalence of primary axillary hyperhidrosis to be around 5.7-5.9% in the population aged 5-64 years, with similar rates observed even in children [5]. This excessive sweat production is not related to the body's need for thermoregulation and can significantly impact an individual's daily life [6]. The symptoms of this condition can lead to considerable discomfort, affecting various aspects of life such as social interactions, work productivity, and overall quality of life [1,2,6,7].

A range of treatment options is available for primary axillary hyperhidrosis, including topical applications, oral medications, botulinum toxin injections, and surgical procedures [2,8]. In Japan, the clinical guidelines recommend botulinum toxin type A and topical aluminum chloride as primary treatments for this condition [9]. More recently, other topical treatments like glycopyrronium tosylate hydrate wipes and sofpironium bromide gel have also been approved for use [2,8]. While various treatments exist, botulinum toxin injections have emerged as a prevalent and effective modality for managing axillary hyperhidrosis [2,10]. These injections have been shown to significantly improve symptoms and are associated with high levels of patient satisfaction [9,11,12]. Notably, in Japan, botulinum toxin type A injections account for a substantial portion, approximately 90%, of the direct medical costs associated with treating axillary hyperhidrosis [9]. However, it's important to note that in Japan, health insurance coverage for botulinum toxin type A in treating primary axillary hyperhidrosis is typically limited to severe cases [9].

It is a common observation that sweating tends to increase during warmer months. While this might suggest a potential seasonal variation in the symptoms of axillary hyperhidrosis, comprehensive research specifically examining the correlation between the demand for treatment, such as botulinum toxin injections, and specific meteorological factors in Japan has been limited [13]. Understanding such a relationship could have significant implications for healthcare resource allocation, patient education, and treatment planning. For instance, if a clear seasonal pattern in treatment demand exists, healthcare providers could anticipate periods of higher need and adjust their services accordingly. Furthermore, patients might benefit from knowing about potential seasonal influences on their symptoms and treatment options.

Based on the Act on Assurance of Medical Care for Older Persons, the Ministry of Health, Labour and Welfare started operating the National Database of Health Insurance Claims and Specific Health Checkups of Japan (NDB) [14]. The NDB data are considered useful in government policymaking regarding the national healthcare insurance system and research because they contain comprehensive data reflecting medical care utilization under Japan's universal health insurance system [14].

This study aims to determine whether there are seasonal differences in the number of axillary hyperhidrosis injections in Japan and to investigate whether meteorological conditions are related to the number of axillary hyperhidrosis injections. The number of axillary hyperhidrosis injections was examined monthly using the NDB database. Additionally, data from the Japan Meteorological Agency were used to investigate correlations between meteorological conditions (temperature, precipitation, all-day solar radiation, wind speed, relative humidity, and atmospheric pressure) and the number of axillary hyperhidrosis injections.

## Materials and methods

This retrospective and descriptive study used NDB open data published and managed by the Japanese Ministry of Health, Labour and Welfare. All investigations adhered to the principles of the Declaration of Helsinki. As the study relied exclusively on an anonymized, publicly accessible database containing no personally identifiable information, ethical review by the institutional review board was deemed exempt.

The number of botulinum toxin type A injections for axillary hyperhidrosis was collected from the NDB database from fiscal years 2019 to 2022. The fiscal month is the same as the actual month. The study period spanned from the fiscal year 2019 (April 2019) to the fiscal year 2022 (March 2023), encompassing a total of 48 months. The procedure code for botulinum toxin type A injection for axillary hyperhidrosis in Japan is G017. We used monthly NDB data from fiscal years 2019 to 2022, as this level of detail became available starting in 2019.

Monthly meteorological data were collected from the Japan Meteorological Agency database [15]. Temperature (°C), precipitation (mm), all-day solar radiation (MJ/m²), wind speed (m/s), relative humidity (%), and atmospheric pressure (hPa) were investigated. Meteorological data were averaged from four major cities (Tokyo, Nagoya, Osaka, Fukuoka) representing the major population of axillary hyperhidrosis injection centers across different regions (Kanto, Chubu, Kansai, Kyushu).

The seasons were divided into spring (March, April, May), summer (June, July, August), fall (September, October, November), and winter (December, January, February) while acknowledging that weather conditions in Japan can be different even during the same season depending on the location [16].

To compare the number of axillary hyperhidrosis injections across the four defined seasons, the Steel-Dwass test was employed. This non-parametric multiple comparison test was chosen as it does not assume normality or equal variances, making it suitable for comparing group medians when assumptions for parametric tests like ANOVA might not be met. To investigate the association between monthly meteorological conditions and the number of axillary hyperhidrosis injections, partial correlation analysis was conducted. This method was selected to assess the relationship between the number of injections and each specific meteorological variable while statistically controlling for the potential confounding effects of the other five meteorological variables included in the model. Statistical significance was set at p<0.05. Statistical analysis was performed using JMP® 16 (SAS Institute Inc., Cary, North Carolina, United States).

In Japan, the first COVID-19 case was identified in January 2020. With the spread of the infection, the Japanese government declared a state of emergency for COVID-19 in April 2020. During the COVID-19 pandemic, non-emergency procedures were postponed; thus, the COVID-19 pandemic may have affected the trends in injections as well. However, this data point was included in the primary analysis to maintain the integrity of the time series while acknowledging its potential deviation.

## Results

**Figure 1:**
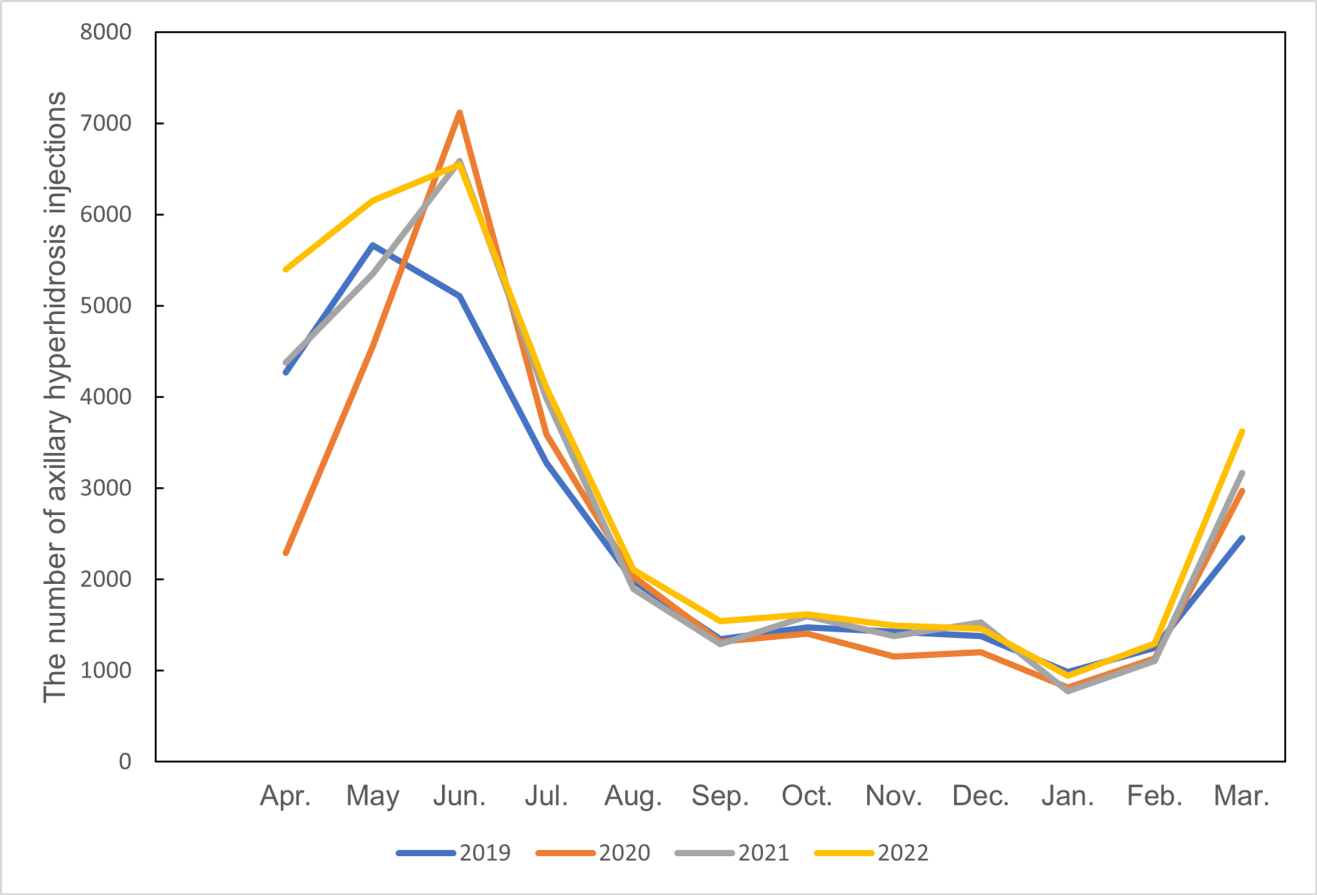
Line graph of the number of axillary hyperhidrosis injections by month from fiscal years 2019 to 2022 The number of axillary hyperhidrosis injections peaked in May and June each year. After June, there is a sharp decline across all years, reaching a stable, lower plateau from August to February. An increase is observed again in March. April 2020 numbers were considered low due to the declaration of a state of emergency during the COVID-19 pandemic.

Figure 1 shows the number of axillary hyperhidrosis injections by month from fiscal years 2019 to 2022. The number of injections peaked in May or June each year. After June, there was a sharp decline across all years, reaching a stable, lower plateau from August to February. An increase was observed again in March. April 2020 numbers were considered low due to the declaration of a state of emergency during the COVID-19 pandemic.

**Figure 2:**
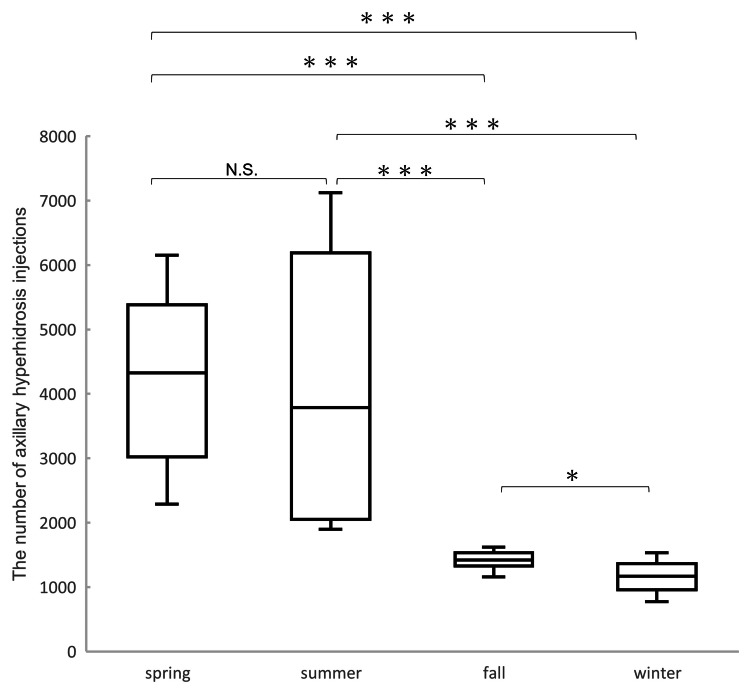
Axillary hyperhidrosis injections box-and-whisker diagram by season from fiscal years 2019 to 2022 Both spring and summer showed markedly higher numbers of injections compared to fall and winter (p<0.001). There was no significant difference between spring and summer (N.S.). A significant difference was observed between fall and winter (p<0.05). *p<0.05; ***p<0.001

The box-and-whisker diagram in Figure 2 shows the number of axillary hyperhidrosis injections by season from fiscal years 2019 to 2022. The number of axillary hyperhidrosis injections varied significantly across the four seasons. Both spring and summer showed markedly higher numbers of injections compared to fall and winter (p<0.001). There was no significant difference between spring and summer (N.S.). Additionally, a significant difference was observed between fall and winter (p<0.05), with fall having more injections.

**Figure 3:**
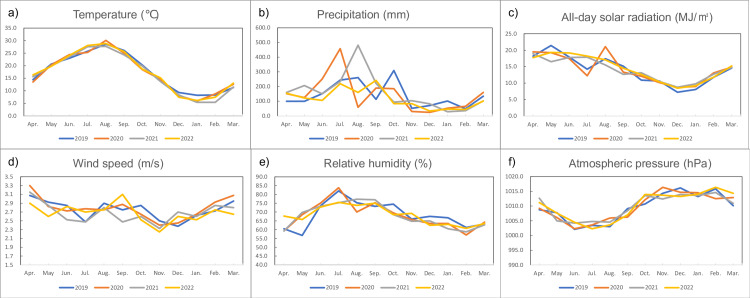
Line graph of monthly meteorological conditions, namely, temperature (°C), precipitation (mm), all-day solar radiation (MJ/m²), wind speed (m/s), relative humidity (%), and atmospheric pressure (hPa), from 2019 to 2022 Monthly meteorological data were collected from the Japan Meteorological Agency database; data are averages for Tokyo, Osaka, Nagoya, and Fukuoka.

Figure 3 shows a line graph of monthly meteorological conditions, namely, temperature (°C), precipitation (mm), all-day solar radiation (MJ/m²), wind speed (m/s), relative humidity (%), and atmospheric pressure (hPa), from 2019 to 2022.

Partial correlation analysis between meteorological data and the number of axillary hyperhidrosis injections is shown in Table 1. All-day solar radiation showed a strong positive correlation (r=0.7193; p<0.0001), while temperature showed a strong negative correlation (r=-0.6052; p<0.0001). Wind speed also showed a significant negative correlation (r=-0.441; p=0.0031). Relative humidity showed a moderate positive correlation (r=0.3626; p=0.0169).

**Table 1:** Partial correlation analysis between meteorological data and the number of axillary hyperhidrosis injections All-day solar radiation showed a strong positive correlation (r=0.7193; p<0.0001). Temperature showed a strong negative correlation (r=-0.6052; p<0.0001). Wind speed also showed a significant negative correlation (r=-0.441; p=0.0031). Strong positive correlations were found with all-day solar radiation, strong negative correlations with wind speed, and weaker or non-significant associations with temperature, precipitation, and atmospheric pressure.

		r	t	p
Axillary hyperhidrosis injections	Temperature	-0.6052	-4.81	<0.0001
	Precipitation	0.1328	0.84	0.3958
	All-day solar radiation	0.7193	6.18	<0.0001
	Wind speed	-0.441	-3.03	0.0031
	Relative humidity	0.3626	2.4	0.0169
	Atmospheric pressure	-0.1848	-117	0.2354
Temperature	Precipitation	0.069	0.44	0.6601
	All-day solar radiation	0.8448	8.86	<0.0001
	Wind speed	-0.364	-2.33	0.0164
	Relative humidity	0.7682	7.08	<0.0001
	Atmospheric pressure	-0.0213	-0.13	0.8923
Precipitation	All-day solar radiation	-0.1501	-0.94	0.3368
	Wind speed	0.5215	3.76	0.0003
	Relative humidity	0.3281	2.13	0.0317
	Atmospheric pressure	-0.0583	-0.37	0.7104
All-day solar radiation	Wind speed	0.5028	3.68	0.0006
	Relative humidity	-0.7114	-6.01	<0.0001
	Atmospheric pressure	-0.2665	-1.71	0.0841
Wind speed	Relative humidity	-0.0459	-0.29	0.77
	Atmospheric pressure	-0.142	-0.9	0.3638
Relative humidity	Atmospheric pressure	-0.3883	-2.6	0.0101

## Discussion

This nationwide study investigated seasonal trends and meteorological correlations in the use of botulinum toxin type A injections for primary axillary hyperhidrosis in Japan, utilizing the comprehensive NDB database from 2019 to 2022. The findings reveal a distinct and reproducible seasonal pattern in treatment demand, as well as notable associations with specific meteorological factors.

The data clearly demonstrate that the number of botulinum toxin injections for axillary hyperhidrosis peaks sharply in late spring and early summer, specifically in May and June, across all years studied. After June, the number of procedures declines rapidly and remains low throughout fall and winter, with only a slight increase observed again in March. Statistical analysis confirmed that both spring and summer had significantly higher numbers of injections compared to fall and winter (p<0.001), while there was no significant difference between spring and summer themselves. Fall also had significantly more injections than winter (p<0.05), but both were much lower than the warmer seasons. This pattern persisted despite the impact of the COVID-19 pandemic, which caused a temporary reduction in procedures during the emergency declaration in April 2020.

Partial correlation analysis between monthly injection numbers and meteorological data provided key insights. All-day solar radiation showed the strongest positive correlation with the number of injections (r=0.7193; p<0.0001), indicating that increased sunlight exposure is closely linked to higher demand for treatment. In contrast, temperature was negatively correlated with injection numbers (r=-0.6052; p<0.0001), a finding that may initially seem counterintuitive. Wind speed also showed a significant negative correlation (r=-0.441; p=0.0031), while relative humidity had a moderate positive correlation (r=0.3626; p=0.0169). Precipitation and atmospheric pressure were not significantly associated with injection frequency.

The observed seasonal peak of botulinum toxin injections for axillary hyperhidrosis in May and June, prior to the hottest months of the year (typically July and August in Japan), suggests that patients may be seeking treatment in anticipation of increased sweating and its social or occupational impact, rather than as a direct response to the highest temperatures. One potential explanation for negative partial correlation with temperature, when controlling for other factors such as solar radiation, lies in the timing of patient behavior and the nature of the treatment. The strong positive correlation with solar radiation suggests that increasing daylight hours and sun intensity in spring (May/June) act as primary cues for patients to seek treatment in anticipation of the upcoming hottest months (July/August). Patients may proactively schedule appointments as they notice longer, sunnier days, aiming to have the botulinum toxin take effect before the peak heat arrives. Consequently, by the time temperatures reach their zenith in July and August, many individuals who intend to seek treatment may have already done so. This would result in a decline in new injection procedures during the period of highest absolute temperatures, despite the underlying trigger (heat and sweating) notionally being at its peak. Furthermore, the administration of botulinum toxin injections for hyperhidrosis has been demonstrated to offer relief for a period of several months. Patients undergoing treatment in May or June are likely to experience sustained efficacy throughout July and August, thereby reducing the immediate requirement for further injections during these months of heightened temperature. This proactive behavior may reflect both an awareness of symptom worsening in the summer and the desire to manage symptoms before they become most disruptive. These findings have practical implications for healthcare providers and policymakers. Recognizing the seasonal surge in demand can help optimize clinic scheduling, staff allocation, and inventory management for botulinum toxin products. Patient education efforts may also be timed to encourage early consultation and treatment planning ahead of the summer months, potentially improving patient outcomes and satisfaction.

In a large-scale survey in Japan reported by Waseda et al. [13], the distribution of the number of patients by time of year and region was investigated for a total of 1,037,269 patients with excessive sweating and other symptoms. The association between the number of patients and meteorological data was analyzed by univariate analysis, and the relevant meteorological data items were reportedly minimum temperature and body surface temperature. While prior studies have established the efficacy and patient satisfaction of botulinum toxin for axillary hyperhidrosis [6,9,11,12,17], few have examined seasonal or meteorological influences on treatment demand [13]. The present findings align with the commonly observed increase in sweating during warmer months but add nuance by highlighting the role of solar radiation and the timing of treatment relative to peak temperatures. This suggests that patient decision-making is influenced by anticipation and external cues, not just symptom severity.

A major strength of this study is the use of the NDB, which covers nearly the entire Japanese population and provides a robust, population-level perspective on treatment trends [18]. The inclusion of multiple years of data and the use of objective meteorological measurements from major urban centers enhance the reliability and generalizability of the findings.

However, several limitations should be acknowledged. First, insurance coverage for botulinum toxin injections in Japan is restricted to severe cases, so the data may underestimate the total number of patients seeking treatment, particularly those with milder symptoms who may use other therapies or pay out of pocket. Second, meteorological data were averaged from four major cities and may not capture regional microclimates or rural-urban differences. Third, the study design is observational and cannot establish causality between meteorological factors and treatment-seeking behavior. The COVID-19 pandemic also introduced an external shock to healthcare utilization patterns, although the overall seasonal trend remained robust [19,20].

Further research should explore the psychological and social factors that prompt patients to seek treatment at particular times, as well as potential regional differences within Japan, as it is difficult to assess the association with patients' medical visitation behavior. Prospective studies could also assess whether the timing of treatment initiation affects clinical outcomes over the summer season. Finally, international comparisons could elucidate whether similar patterns exist in other countries with different climates and healthcare systems.

## Conclusions

The use of botulinum toxin injections for primary axillary hyperhidrosis in Japan exhibits a clear seasonal pattern, peaking in late spring and early summer. This trend is most strongly associated with increasing solar radiation rather than temperature alone, suggesting that patients seek treatment proactively as summer approaches. These insights can inform resource planning, patient education, and policy decisions to better meet the needs of individuals with axillary hyperhidrosis.
